# Mississippi Valley Association of Dental Surgeons

**Published:** 1855-07

**Authors:** A. M. Leslie


### Dental Register of the West

VOL. VIII. JULY, 1855. NO. 4.

### Proceedings of Societies.

### MISSISSIPPI VALLEY ASSOCIATION OF DENTAL SURGEONS.

Special meeting held May 7th to receive the members of the American Society, persuant to resolution adopted at the annual meeting.

Present Drs. TAFT, WATT, WEBSTER, GODDARD, HOW, TAYLOR, SMITH, MCCULLOM, BROWN, BONSALL and LESLIE.

The President and Vice-President being absent, on motion of Dr. Goddard, Dr. Bonsall was called to the chair.

On motion, Dr. Watts, chairman, presented the following report.

REPORT

*To the Mississippi Valley Association.*

Your committee to whom was referred the prize essay would respectfully report that though unable to act in concert to the extent they desired, yet they have endeavored to carry out the wishes of the association, as well as they could ascertain them from the remarks of members at the last meeting.

The whole essay has been revised and verbal corrections have been made in several places. But our attention was principally directed to the chapters objected to and these are very materially modified, and, as now presented, will, we trust, meet the views of the society. The articles on Extraction, and Mechanical Dentistry are much abridged, and we invite the special attention of the society to them and to the one on "Filing the teeth." The work is reduced about twelve manuscript pages.

We would therefore recommend the adoption of the following resolution:

*Resolved*, That a committee of Three be appointed to publish —— copies of the essay and have the same ready for distribution at the next annual meeting of the association.

Respectfully submitted

G. WATT,
J. TAYLOR.
J. TAFT.

Report accepted, and on motion, laid over until next day.

Dr. Goddard moved, that when we adjourn, we do so to meet at 8½ o'clock to-morrow. Adopted.

The following communication was presented by the president.

*To the President of the M. V. A. D. S.*

Sir, I hereby resign my place on the committee on "Dental Progress." GEO. WATT.

Dr. Gooddard moved to lay on the table until to-morrow. Carried.

On motion adjourned.

SECOND DAY 8½ O'CLOCK A. M.

On motion, the following resolutions were unanimously adopted.

*Resolved*, That the members of the Mississippi Valley Association of Dentists and of the profession generally of the West cordially extend to the members of the American Society, and our brethren who are now visiting the city of Cincinnati an invitation to meet us on Wednesday evening, May 9th, at the Walnut Street House to participate with us in a social entertainment.

*Resolved,* That a committee of Three be appointed to make all necessary arrangements and extend such invitations as they deem proper, and report as soon as such arrangements are completed.

Dr. Goddard moved that the amended portions of Dr. Watt's essay be now read. Adopted.

*Resolved,* That 300 copies of the essay be published, in the cheapest form, and be distributed by the committee to those members of the profession for amendments and criticism, as they may deem proper, with a request that they will suggest such amendments to the committee, as they may deem advisable, as soon as they can conveniently—said committee to report at our next meeting.

*Resolved,* That the committee on the foregoing essay be instructed to carry it out.

Dr. Goddard moved that the resignation of Dr. Watt be laid on the table indefinitely. Adopted.

By order of the society the name of Dr. E. A. Herman which was left out, by the action of the society at our last meeting, be restored—Dr. J. Taylor having reported that his initiation fee had been paid him previous to our last meeting.

Moved that, when we adjourn, we do so to meet at 4 o'clock P. M.

Dr. Gooddard moved that in the absence of the president Dr. Taylor be appointed to welcome the American Society in the name of this association, and that Dr. Watt be a committee to invite said society to hold their sessions in this hall.

The members of the American Society being introduced Dr. Taylor welcomed them to the West and pledged a united effort to make their visit a pleasant one. When on motion the society adjourned.

AFTERNOON SESSION.

Committee on entertainment reported. Report accepted. Adjourned.

At 9 o'clock P. M. The society meet at the Walnut Street Hotel where with the members of the American Society together with a number of invited guests Medical and Dental to the number of about fifty, they spent two hours pleasantly around the social board. In answer to toasts, speeches, short but pithy, were made by Drs. Townsend, Taylor, Dunning, Allen, Jobson and Miller of the dental profession, and by Professors Wood, Armor and Mendenhall of the Medical.

A very pleasant social evening was also enjoyed on the succeeding night at the hospitable board of our worthy ex-president Dr. Chas. Bonsall.

The members of the M. V. Association and others who enjoyed the society of their eastern brethren will recall with pleasure their visit.

A. M Leslie, Sec'y.

